# Nutritional Therapy in Liver Transplantation

**DOI:** 10.3390/nu9101126

**Published:** 2017-10-16

**Authors:** Ahmed Hammad, Toshimi Kaido, Vusal Aliyev, Claudia Mandato, Shinji Uemoto

**Affiliations:** 1 Division of Hepato-Biliary-Pancreatic and Transplant Surgery, Department of Surgery, Graduate School of Medicine, Kyoto University, Kyoto 606-8501, Japan; ahmedhammad2005@yahoo.com (A.H.); dr.vusal@outlook.com (V.A.); uemoto@kuhp.kyoto-u.ac.jp (S.U.); 2 Department of General Surgery, Mansoura University, Mansoura 35516, Egypt; 3 L’AORN Children’s Hospital Santobono and Pausilipon, Napoli 80122, Italy; cla.mandato@gmail.com

**Keywords:** liver transplantation, immunonutrition, synbiotics, nutritional intervention, sarcopenia, branched-chain amino acids, nutraceuticals

## Abstract

Protein-energy malnourishment is commonly encountered in patients with end-stage liver disease who undergo liver transplantation. Malnutrition may further increase morbidity, mortality and costs in the post-transplantation setting. The importance of carefully assessing the nutritional status during the work-up of patients who are candidates for liver replacement is widely recognized. The metabolic abnormalities induced by liver failure render the conventional assessment of nutritional status to be challenging. Preoperative loss of skeletal muscle mass, namely, sarcopenia, has a significant detrimental impact on post-transplant outcomes. It is essential to provide sufficient nutritional support during all phases of liver transplantation. Oral nutrition is preferred, but tube enteral nutrition may be required to provide the needed energy intake. Herein, the latest currently employed perioperative nutritional interventions in liver transplant recipients are thoroughly illustrated including synbiotics, micronutrients, branched-chain amino acid supplementation, immunonutrition formulas, fluid and electrolyte balance, the offering of nocturnal meals, dietary counselling, exercise and rehabilitation.

## 1. Introduction

The liver is the largest and most crucial metabolic organ, playing a pivotal role in integrating several biochemical pathways of carbohydrate, fat, protein, and vitamin metabolism as well as the transport of lipids and the secretion and excretion of bile, all of which are processes involved in muscle and protein metabolism and central for well-nourished status [1,2,3].

Advances in post-transplant care and management of graft rejection have greatly improved outcomes for patients after orthotopic liver transplantation (LT). However, malnutrition is a relevant factor when determining the progress of hepatic disease, as it contributes to hypoalbuminemia and intensifies the hydroelectrolytic imbalance determined by renal alterations [3,4,5,6,7].

Protein energy malnutrition (PEM) is a common problem in patients with end-stage liver disease (ESLD) awaiting LT [5]. This applies to nearly every etiology of ESLD with the exception of fulminant hepatic failure. The diagnosis of PEM in ESLD is established by marked muscle wasting and subcutaneous fat loss [5,6]. PEM is more prevalent in those with decompensated liver disease i.e., those with ascites, portosystemic hepatic encephalopathy (HE), portal hypertensive bleeding and patients hospitalized for alcoholic liver disease than in patients with nonalcoholic liver disease [7].

Clinical features of deteriorated liver function tend to normalize following successful LT. However, PEM can significantly increase the operative risk at the time of surgery and is a risk factor for morbidity, short- and long-term mortality in patients undergoing LT [7,8] and decreased graft survival after LT [9]. Moreover, PEM predisposes a patient to complications such as compromised respiratory function, wound-healing problems, longer dependency on mechanical ventilation, increased rates of septic complications, use of antibiotics, blood products usage, length of hospital stay, intensive care unit admissions, delayed physical rehabilitation, with substantially higher cost of the transplant [8,9]. Prolonged waiting times worsen outcomes when patients are already malnourished [10].

A patient’s nutritional status can worsen rapidly in the immediate postoperative period due to perioperative malnutrition, surgical stress, immunosuppressive therapy, post-interventional complications, postoperative protein catabolism, and fasting periods [5,6]. This suggests the need for preemptive nutritional support with liver-adapted formulas containing additional carbohydrates, fat and proteins especially branched-chain amino acids (BCAAs) [7,8].

Body cell mass (BCM) is defined as the sum of intracellular water and fat-free mass, including skeletal muscle and viscera, without bone mineral mass. BCM comprises the metabolically active and protein-rich compartments in the body responsible for basal energy expenditure (BEE) and is known to be depleted in patients with PEM [9]. Loss of skeletal muscle mass (SMM) or sarcopenia, reflecting preoperative malnutrition is a common complication of liver cirrhosis (LC). Sarcopenia could interfere with early postoperative mobilization and has been found to be closely associated with post-LT mortality in patients undergoing living donor LT (LDLT) [9]. Sarcopenia confers a vulnerability to preoperative infection, including spontaneous bacterial peritonitis, pneumonia, post-LT bacteremia, sepsis and wound dehiscence and pulmonary complications, including aspiration pneumonia and atelectasis secondary to deteriorated immune functions [10,11]. Although less apparent, muscle wasting may be present in obese patients, which is identified as “sarcopenic obesity” [10,11] and underscores the importance of considering muscle depletion in such patients. This observation is relevant because the number of obese patients, with ESLD due to Non-alcoholic steato-hepatitis, is increasing among those awaiting LT [11,12].

An aggressive approach to ensure adequate nutritional repletion, as well as correcting vitamin and micronutrient deficiencies is central to maintain remaining hepatic function, improve the patient’s metabolic reserves, and the outcomes after LT [1]. Hence, timely nutrition assessment and patient tailored intervention for anticipated recipients or those on the waiting list may improve outcomes surrounding LT [1,2,3].

### 1.1. Etiology of Malnutrition

#### 1.1.1. Decreased Energy Intake

Patients with liver disease have decreased energy intake due to anorexia caused by zinc deficiency, hyperglycemia and increased pro-inflammatory cytokine levels; tumor necrosis factor-alpha (TNF-α), interleukin (IL)-6 and leptin [12,13,14,15]. Other causes of loss of appetite in those patients are the unpalatable specialized diets; low-salt and low-protein diets for ascites and HE, respectively, and altered gustatory sensation due to hypomagnesemia and autonomic neuropathy in LC, which also causes gastroparesis and delayed bowel transit time, together with conditional bacterial overgrowth on top of tense ascites, early satiety due to less gastrointestinal peristalsis and gastric restrictive expansion due to large volume ascites, maldigestion, impaired hepato-intestinal extraction, malabsorption and intestinal mucosal atrophy associated with portal hypertensive gastroenteropathy [12,13,14,15,16]. Increased protein losses due to gastrointestinal bleeding and frequent large volume paracentesis also contribute to malnutrition, which may further be worsened by protein-losing enteropathy, exacerbating hypoalbuminemia. In liver cirrhosis, up to 45% of patients may have a coexisting infection with *Helicobacter pylori* may cause dyspepsia and a decreased desire for food [13]. Moreover, limited intake due to diagnostic procedural events can lead to prolonged nil per os may further contribute to PEM [14,15,16,17].

#### 1.1.2. Hypercatobolic Status

A number of cirrhotic patients may also have increased BEE that may be related to increased sympathetic nervous system activity and inflammation. Fever, spontaneous peritonitis and bacterial translocation are undoubtedly considered as the most common inducing factors contributing to accelerating catabolism [18]. Although hyperinsulinemia is present, glucose intolerance ensues due to insulin resistance, decreased glycogen stores and impaired glycogenolysis. This subsequently leads to gluconeogenesis from lipid peroxidation and mobilization of amino acids from the skeletal muscles and visceral proteins demonstrating muscle depletion and decrease in subcutaneous fat [17,18,19].

Patients with protein malnutrition have increased protein requirements in order to maintain a positive nitrogen balance [19,20]. Low plasma levels of insulin growth factor (IGF-1), which mediates most of the growth-promoting effects of growth hormone, also explain the severe growth hormone resistance seen in patients with ESLD [20].

Moreover, patients with ESLD may have impaired synthesis of polyunsaturated fatty acids from their essential fatty acid precursors with increases in levels of *n*-6 and *n*-9 fatty acids and decreases in *n*-3 moieties in plasma and adipose tissue [21]. Enhanced gluconeogenesis, especially after fasting, with preference for fat metabolism, lipid peroxidation and lipolysis, also increases upon impaired glycogen storage and utilization [22].

#### 1.1.3. Decreased Nutrient Absorption

**Figure 1:**
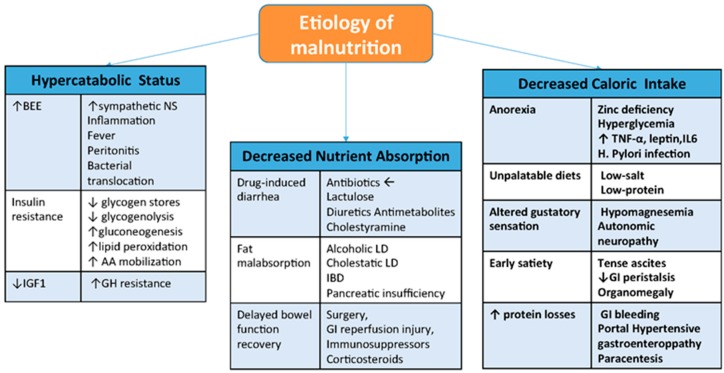
Etiology of Malnutrition in End Stage Liver Disease. BEE: basal energy expenditure; IBD: inflammatory bowel disease; IGF1: insulin growth factor 1; IL6: interleukin-6; GI: gastrointestinal tract; GH: growth hormone; LD: liver disease; NS: nervous system; TNF-α: tumour necrosis factor alpha.

Other contributing factors include drug-induced diarrhea (like neomycin, lactulose, diuretics, antimetabolites, cholestryamine) [12,23]. Fat malabsorption in alcoholic and cholestatic liver diseases, especially sclerosing cholangitis with inflammatory bowel disease or concomitant pancreatic insufficiency can result in specific fat-soluble vitamin deficiencies [23]. Also, metabolic stresses from surgery, gastrointestinal reperfusion injury, immunosuppressive therapy and corticosteriods use lead to delayed bowl function recovery and disorder of nutrients absorption (Figure 1).

### 1.2. Assessment of Nutritional Status

All patients being prepared for LT should undergo a thorough nutritional evaluation. Traditional assessment tools as BMI and anthropometry are not accurate in patients with liver disease due to fluid retention and organomegaly found in a significant number of patients. No gold-standard evaluation exists to determine the extent of malnutrition in patients with ESLD [6,24].

#### 1.2.1. Subjective Global Assessment

The initial assessment should begin with a careful history to document weight loss, nausea, anorexia, and specialized diets and supplements. A complete physical examination should search for changes in oral mucosa, skin and hair, loss of subcutaneous fat, muscle-wasting and stigmata of chronic liver disease. Subjective global assessment (SGA) combines a thorough history-taking and physical examination and rates patients either “well-nourished”, “moderately malnourished”, or “severely malnourished” [6]. This test has shown high specificity with very low sensitivity for diagnosing malnutrition in patients with alcoholic liver disease. However, it has not been found to be a reliable tool for evaluating nutritional status in LT patients [6,24]. First, SGA depends on personal information that can be difficult to obtain from patients with cognitive impairment or somnolence. Second, the only anthropometric measure utilized is the body weight, which is often changed by ascites and edema [6,24].

#### 1.2.2. Biochemical Parameters and Immune-Competence

Biochemical tests used as nutritional indicators include serum transferrin and retinol binding protein levels [25,26]. A reduced level of serum transferrin is additionally indicative of decreased energy protein intake related to LT patients [25]. However, such parameters have not been shown to be accurate indices of nutritional status given the fact that their levels correlate with severity of liver damage and inflammatory states rather than malnutrition due to the catabolic nature of liver disease and associated protein turnover.

Serum albumin frequently serves as an important indicator of liver function. However, it has a long half-life (17–21 days) and because exogenous albumin supplements are frequently administered in clinical practice, serum albumin levels cannot sensitively or dynamically reflect early liver damage. The shorter half-life (2–3 days) of prealbumin (transthyretin) renders it a more sensitive indicator of damaged synthesis functioning in the liver and fluctuations in nutritional status than albumin [27]. Creatinine-height index, 24-h urine nitrogen and 3-methylhistidine excretion were suggested as indirect measure of body muscle mass, as 1 g of excreted creatinine equals 18.5 kg of muscle mass [26].

Immune competence, which is considered a functional test of nutritional status, may be affected by hypersplenism, abnormal immunologic reactivity and alcohol abuse [26]. Low total lymphocyte, CD8 cell counts, and abnormal response to skin anergy tests; delayed cutaneous hypersensitivity response (≥5 mm induration to two or more skin tests) were suggested parameters for malnutrition [27]. The measurement of lean cell mass with total body potassium has been postulated for nutritional assessment of children but it is not a part of routine nutritional assessment of children [28].

#### 1.2.3. Anthropometry

Anthropometric measurements, such as body mass index, mid-arm muscle circumference, triceps skin fold thickness, biceps or subscapular skin fold thickness are simple, quick, cheap and noninvasive assessment methods in patients with liver disease [25,26,27,29]. However, these measures have been questioned regarding their reliability in patients with ascites and peripheral edema but correction by subtracting estimated amounts of ascites and other fluid collections may compensate for this disadvantage to some extent [25,26,27,29]. Triceps skinfold thickness remained inadequate in 70% of malnourished patients at the end of the first year [30]. In a prospective cohort study, cirrhotic patients who were severely malnourished while on the waiting list showed further deterioration at 3 months after transplantation; however, they improved at 6 and 12 months. Once again, primary changes were observed for fat mass (median triceps skinfold: basal 10.8 mm vs. 15.2 mm, 12 mm *p =* 0.03) [30].

#### 1.2.4. Hand Grip Dynamometry

Functional assessment such as handgrip strength dynamometry and the six-minute walk test have been advocated as potential good parameters not only to assess but also to follow up nutritional interventions [27]. Hand grip strength is good reflector of upper limb strength and can used serially in conjunction with bioelectrical impedance analysis (BIA) to follow up adopted nutritional therapy regimen [29,30,31,32].

#### 1.2.5. Indirect Calorimetry

The BEE can be predicted with several formulas such as the Harris-Benedict or Schofield equations [33]. The REE is an estimated value of BEE accounting for the effects of food, temperature, stress and activity factors [19,30]. The non-protein respiratory quotient (npRQ), a unitless number measured by 24 h urinary urea nitrogen, body carbon dioxide production and oxygen consumption was used to evaluate the nutritional status of patients with LC [34]. Moreover, npRQ provides useful information regarding substrate utilization in the post-operative period which can be very helpful in the development of a nutrition support strategy [34].

#### 1.2.6. Bioelectrical Impedance

In nutritional assessment for patients undergoing LDLT, Kaido et al. [10,11,12] used direct segmental multi-frequency BIA with the InBody720 (Biospace, Tokyo, Japan) device, by which body mass index, intra- and extracellular water, and body fat percentage could be automatically and measured within 2 min. SMM was measured and shown as a percent against standard SMM calculated by sex and height of each patient. BCM was automatically calculated by the InBody 720 and displayed as a normal range (e.g., 23.0–28.1 kg).

The BIA measures the body’s resistance to flow (impedance) of alternating electrical current at a designated frequency between points of contact on the body. Water in body tissue is conductive; therefore, BIA can indirectly provide information on the body’s tissue content including total body water, fat-free mass, and SMM [7,9]. BIA is increasingly being used because it is easy to perform, portable, non-invasive, and quick despite some limitations in patients with ascites. It has been highly correlated with hydrostatic weighing, dual energy X-ray absorptiometry, in vivo-neutron activation analysis, and deuterium isotope dilution, without the radiation exposure hazards [35,36,37,38]. BIA might be considered comparable to measuring the psoas muscle cross sectional area at the L_3_ vertebral level by computed tomography (CT) scan or magnetic resonance imaging (MRI) as it might be appropriate to evaluate not only the psoas muscle mass, but also the whole body SMM [39,40,41].

**Figure 2:**
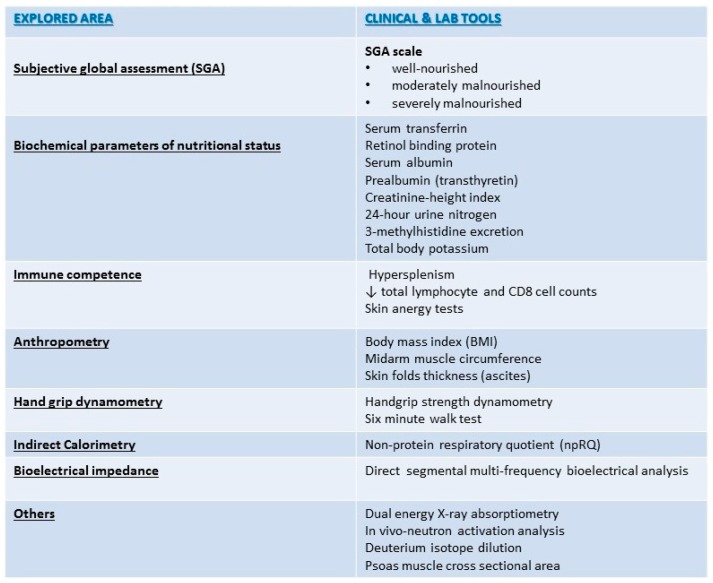
Assessment of nutritional status in End Stage Liver Disease Patients.

Considering feasibility, the current guidelines of the European Society for Clinical Nutrition and Metabolism (ESPEN) recommend only SGA and/or anthropometry parameters to identify patients at risk for poor nutritional status, and BIA to quantify undernutrition in spite of the limitations of all techniques in patients with ascitic decompensation [40,41,42]. According to the ESPEN, other composite nutrition scores provide no additional prognostic information [42,43,44] (Figure 2).

## 2. Nutritional Therapy before Liver Transplantation

The main goals of pre-LT nutritional therapy are to prevent further nutrient and muscle depletion and to correct any vitamin and mineral deficiencies present in order to minimize the risk of infections and debility [23].

Because in deceased donor transplantation (DDLT), no one can predict when a patient will receive a transplant [2], an aggressive early post-operative nutritional support (by enteral route if possible) should be allocated to those patients with highest MELD scores, especially when they are undernourished and, if it is anticipated that patients will be unable to eat within for more than two days. Also, this approach should be considered when patients cannot maintain oral intake above 60% of the recommended intake for more than 10 days [18]. On the other hand, an early, planned, preoperative nutritional intervention can be performed in most cases of LDLT, since the date of LT is known in advance, unlike in DDLT. Nutritional therapy, as well as rehabilitation at the time of referral of a potential recipient, starts approximately a few months before LT to most effectively increase SMM and BCM [7].

### 2.1. Route of Nutritional Support

Enteral nutrition (EN) with a gastric or jejunal small-bore feeding tube is the preferable route of delivery of nutrition for all patients who are not able to maintain adequate oral intake to still benefit from topical nutritional factors in the gut, and maintain the integrity of the gastric mucosa and gut barrier. It is also less costly, with fewer complications and decreased hospital length of stay compared with parenteral nutrition (PN) which carries risk of infection, fluid overload and electrolyte imbalance [45]. ESPEN guidelines (2006) on enteral nutrition in organ transplantation recommended use of nutritional support in patients with severe nutritional risk for 10–14 days prior to major surgery even if surgery has to be delayed [41,42].

EN provides antigenic stimulation to the gut-associated lymphoid tissue and is a stimulus for biliary secretion of immunoglobulin A [10,46]. These factors maintain the barrier against translocation of luminal bacteria to the portal circulation, thus decreasing infectious complications indicated by the differential urinary excretion of carbohydrates of varying molecular weights [10,47].

With EN, excessive feeding will lead to intolerance with diarrhea, bloating, and vomiting. Because the gut provides a “gate-keeper” role, major complications related to excessive tube feed administration are generally kept to a minimum. With PN however, there are no means of regulation and the patient is forced to assimilate the entire substrate load [10,45].

Feeding tubes do not increase the risk for esophageal variceal hemorrhage, but may be associated with an increased risk of epistaxis, sinusitis, impaired gastric emptying and tube feeding-associated diarrhea on long-term use as well as tube retraction, clogging and small intestinal obstruction. However, complications related to mal-positioned feeding tubes are usually preventable if placement is correctly achieved and regularly monitored [10,47].

Feeding tubes are preoperatively placed endoscopically by transillumination across the abdominal wall. The presence of ascites may increase the difficulty in finding a safe window for tube placement. Fluid in the peritoneal cavity can prevent proper apposition of the gastric body to the abdominal wall to ensure the healing of a proper tube tract. Ascites that accumulates can also drain through the tract and increase the risk of infection. Particularly in the setting of hypocomplementemia in cirrhosis, the decreased opsonization and the open communication from the skin to subcutaneous tissue and ascitic fluid could further increase the risk of bacterial peritonitis. The concern is for puncture of a variceal vessel during the procedure. In addition, portal hypertension can lead to many small collateral vessels subcutaneously that also elevate bleeding risk [18,43].

The indications for PN use in liver disease have recently been reviewed and published by ESPEN for patients with fulminant hepatic failure and coma, and for patients who are moderately or severely malnourished and cannot achieve adequate energy intake, either orally or through EN due to gastrointestinal dysfunction such as esophageal bleeding, ileus or intestinal obstruction or those who should be [41,48]. Given the low glycogen stores in patients with liver disease, it is important to provide a glucose infusion in patients who require fasting and are not able to take oral nutrients or EN for more than 12 h [41,42]. Use of “standardized” formulas should be restricted to stable patients with no fluid overload who need maintenance fluid administration only [48]. As for stable patients, the option exists to use fluid maintenance containing water, electrolytes, water- and fat-soluble vitamins or the liver-adapted solutions containing higher BCAA and lower content of aromatic amino acids [49].

### 2.2. Considerations for Carbohydrate Supplementation

Carbohydrate intake should exclusively be provided by glucose and cover 50–60% of non-protein energy requirements. Glucose infusion should supply 2–3 g/kg body weight per day of glucose. Administration of glucose in excess will result in severe hyperglycemia, lipogenesis and increased carbon dioxide production [47,48]. Patients with liver failure can have alterations in glucose homeostasis; therefore, careful monitoring of serum glucose is needed to avoid complications associated with hyperglycemia [48]. In the early postoperative phase, a dysfunction of glucose metabolism associated with insulin resistance is often prevalent. Increased ischemia-related damage of neurons and glia cells, dysfunctional leukocyte function or oxidative stress have been found to be associated with hyperglycaemia. Thus, hyperglycaemia should be treated by reducing the glucose intake to 2.0 g/kg/day, and up to 4 IU/h insulin, if needed, to maintain euglycaemia because higher insulin doses do not improve glucose oxidation. When tacrolimus is used for immunosuppression, its diabetogenic potential can be lowered by reducing the dose and aiming for trough levels of 3–8 ng/mL without undue risk of rejection [49].

### 2.3. Considerations for Lipid Supplementation

Patients with advanced LC have decreased plasma levels of essential fatty acids and their polyunsaturated derivatives such as arachidonate levels that have been associated with lower survival. Both are cell membrane components and precursors of a wide array of biologically active compounds. Lipid should be provided by using emulsions with a reduced content of polyunsaturated fatty acids, as compared to pure soy bean oil emulsions, and cover 40–50% of non-protein energy requirements [22,50].

Because fat is important in nutrient repletion of the malnourished patient, dietary fat should not be restricted unless true fat malabsorption has been diagnosed using a fecal fat test. Incorporating medium chain triglycerides; an alternative form of fat not requiring bile salts for absorption, can provide a concentrated source of calories to patients with fat malabsorption and are available in both EN and PN formulations [22,50].

Many EN formulas provide a wide range of lipid dosages from a variety of sources for fatty acids. When prescribing total parenteral nutrition (TPN) many hospitals compound “three-in-one” TPN solutions containing amino acids, dextrose, and lipids. The minimum lipid dose in such combinations should be 20 g/L or 2% final concentration. More dilute lipid formulas are unstable in the presence of hypertonic dextrose and amino acids, resulting in separation of the lipid emulsion into oil and water [50]. However, Clinical essential fatty acid deficiency takes approximately 5 to 6 weeks to develop without linoleic acid or linolenic acid intake, so it is not likely to become an issue for most patients with liver failure except in those severely malnourished. Therefore, a short course of “fat-free” TPN might be used [22,51].

Other hospitals favor separate “piggyback” lipid infusion which may be preferred due to increased infection risk that comes from TPN solutions hanging for up to 24 h and the use of multi-dose lipid vials for compounding TPN [40,52]. A large dose of PN lipid can result in reticuloendothelial system blocking which aggravates infection risk and is exacerbated by rapid “piggyback” infusion techniques, and ameliorated by slower continuous infusion. Lipid administration should not exceed 1 g/kg/day using pre-hospital dry weight and should be given over 24 h if possible [49,52].

The use of an omega-3 fatty acid-predominant lipid emulsion can prevent the occurrence of dietary-induced and parenteral nutrition-induced steatosis and improves the resolution of cholestasis. Omega-3 can decrease de novo lipogenesis, interfere with the arachidonic acid pathway of inflammation thereby reducing the availability for eicosanoid-synthesizing enzymes and inflammation. It is thought that appropriate intake of omega-3 fatty acids would improve immunological resistance and offer some protection against inflammatory tissue damage and capillary permeability [52,53]. 

### 2.4. Considerations for Protein Supplementation

The dilemma of sarcopenic chronically malnourished hepatopathic patients with ESLD and cirrhosis, having a subtle border between the need for hypercaloric diets rich in proteins and the risk of hyperammoniemia and HE is still to be solved. In these patients, the need for severe protein restriction, however, may be alleviated by measures, such as lactulose and neomycin or probiotics to decrease intestinal ammoniogenesis and BCAAs [54].

Hyperammonemia results from the production of ammonia in the gut and kidneys and the decreased breakdown by liver and skeletal muscle, caused by sarcopenia in malnourished patients with liver disease. It is well known that ammonia has a direct toxicity on brain astrocytes. This effect definitely contributes to HE. In addition, inflammation, infection, and oxidative stress also play a role [54].

Protein intake should not be limited as this may aggravate protein deficiency, and improvement in nitrogen balance may be achieved without aggravating HE [55]. Supplementation with vegetable, rather than animal, source protein may be advantageous [56].

In practice, whole-protein formulas are generally recommended, and BCAA-enriched formulas should be used in patients who develop HE during re-feeding. Protein intake should be at least 1 g/kg/day initially, and then 24-h urinary urea nitrogen can be measured to assess the catabolic rate in patients with normal renal function. Further increases in protein intake can be adjusted accordingly. Progressive increments in protein supplementation should be implemented, up to 1.8–2.0 g/kg/day as tolerated [52,56].

### 2.5. Branched-Chain Amino Acid Supplementation

**Figure 3:**
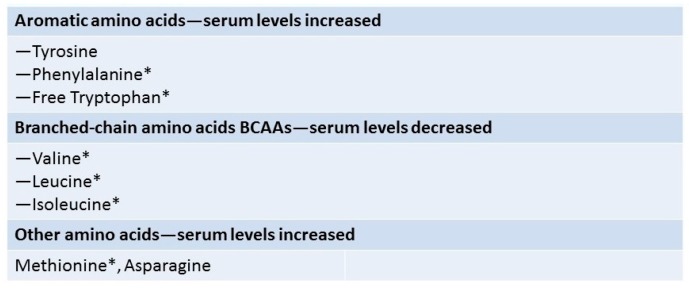
Amino Acids Altered in Liver Disease. The expected BCAAs/tyrosine ratio (Fisher’s ratio), should be 3.5:1; it falls to 1:1 in patients with end stage liver disease. * Essential amino acids.

BCAAs (leucine, isoleucine and valine) do not require the liver for metabolism, and thus are preferentially used in liver failure. On the other hand, aromatic amino acids (AAAs) (phenylalanine, tryptophan and tyrosine) are not metabolized effectively in liver failure and thus accumulate [52,53,54,55,56,57]. The expected ratio, the so-called Fisher’s ratio, or the BCAAs/tyrosine ratio (BTR) should be 3.5:1; however, this ratio falls to 1:1 in patients with ESLD, allowing preferential transport of the AAAs to occur across the blood-brain barrier [58]. These are metabolized to octopamine, phenylethylamine, and phenylethanolamine, which are weak false neurotransmitters that compete with endogenous neurotransmitters, inhibit excitatory stimulation of the brain, competing with endogenous neurotransmitters, thus aggravating HE [57,58]. In addition, tryptophan is metabolized to 5-hydroxytryptophan (serotonin), which can produce further lethargy [55,56] (Figure 3).

There is a debate on the use of BCAA-enriched versus standard amino acid formulas [59,60,61] based on the hypothesis that decreased BTR contributes to HE [62]. However, ESPEN guidelines do not recommend using specialized formulas [41,42].

BCAAs induce secretion of hepatocyte growth factor and glutamine production [63,64,65,66]. Leucine activates the mammalian target of rapamycin signaling pathway, thus inhibiting protein degradation and activating glycogen synthase [7,11].

Shirabe et al. [67] reported preoperative oral BCAA supplementation reduced the incidence of post-transplant bacteremia and sepsis in LDLT patients. Nakamura et al. [68] reported that the phagocytic functions of neutrophils and killer lymphocytes obtained from LC patients were restored by oral BCAAs supplementation.

Recently, a pre-LT BCAA-enriched formula has been demonstrated to lower ammonia, and improve albumin, prealbumin, total lymphocyte count, BTR, glucose intolerance, liver regeneration, immune system function, maturation of dendritic cells and the ability of peripheral blood mononuclear cells to proliferate in response to mitogens, thus preventing postoperative sepsis [7,65,67].

Initiation of oral BCAAs in patients in the early stage of liver disease may contribute to solving current LT problems, such as the donor shortage, the availability of only small liver grafts for patients awaiting LDLT. The use of oral BCAAs might also play a role in improving post-LT mortality by preserving the hepatic reserve of scheduled LT recipients [68,69].

BCAA supplementation post-exercise attenuated the decline in myofibrillar protein synthesis, which is vital in preserving lean mass during weight loss. Thus, the addition of BCAA supplements may have allowed for the maintenance of lean muscle mass because of its potential to enhance lean muscle protein synthesis [65,70]. Oral ingestion of a BCAA supplement before or after exercise improved the recovery of damaged muscles by suppressing the endogenous muscle-protein breakdown [64,66,70].

### 2.6. Micronutrient Supplementation

Patients with ESLD are susceptible to severe deficiencies in folate and pyridoxal-5′-phosphate, the biologically active vitamin B6. Thiamine liver stores are depleted in alcoholic and hepatitis C-related LC [71,72,73]. This depletion is associated with increased brain ammonia concentrations due to decreased activity of a-ketoglutarate dehydrogenase, a rate-limiting tricarboxylic acid cycle enzyme [71,72]. Deficiencies in antioxidant micronutrients (selenium, vitamin E, vitamin C) are related to oxidative stress common in such patients [73].

A typical feature of alcoholic or cholestatic liver diseases is an increasingly severe reduction in hepatic vitamin A stores, which sometimes leads to infertility and night blindness [73,74,75]. In vitamin A-deficient cirrhotic patients, the supplementation of vitamin A, even at relatively moderate doses, may further aggravate liver injury since high-dose vitamin A preparations may be hepatotoxic due to polar retinoid metabolites that cause hepatocellular apoptosis and may promote fibrogenesis [74,75]. Decreased levels of folate, B12, calcium, phosphorus and vitamin K with subsequent coagulopathy are also common [75,76,77,78].

Magnesium and zinc deficiency are common in patients with decompensated LC due to decreased absorption and diuretic-induced increased urinary excretion [31,32]. Clinically, zinc deficiency presents with alterations of smell and taste, alterations in protein metabolism, and HE. Zinc supplementation improves glucose intolerance and decreases ammonia levels [31,32,76].

Bitetto et al. [79] observed that vitamin D may act as an immune modulator in LT, favoring immune tolerance of liver allograft. Additionally, Bitetto et al. found that early vitamin D supplementation, in addition to preventing osteoporosis, was independently associated with a lack of acute rejection, which is important because low vitamin D levels are prevalent among LT candidates.

On the other hand, an excess of micronutrients can also be dangerous. Serum ferritin is associated with increased body iron or can be a consequence of systemic necro-inflammatory states. The level of serum ferritin acts as a predictor of mortality in LC patients [77].

### 2.7. Correction of Liver Osteodystrophy

Osteopenia and osteoporosis are highly prevalent in patients with ESLD, and represent a major cause of morbidity before and after LT [80]. They can be caused by hormonal changes in parathormone and calcitonin, increased circulating levels of bilirubin and cytokines, corticosteroids, and immunosuppressive therapy in cholestatic diseases [81,82,83]. Ingestion of alcohol can directly and indirectly promote bone loss. However, poor diet, physical inactivity, and degree of liver insufficiency further contribute to deterioration of bone [81,82].

LT candidates should be encouraged to consume foods high in calcium and vitamin D. If consumption is low, calcium and vitamin D supplementation (1200–1500 mg/day) is highly recommended for patients with osteopenia and in combination with bisphosphonates for patients with established osteoporosis and/or history of fractures. If steatorrhea is diagnosed, as in cholestatic diseases water-miscible forms of fat-soluble vitamins including vitamin D should be prescribed [81].

Protein metabolism generates a large amount of acid, which must be buffered by the skeleton and kidneys. The skeleton responds to high serum acidity by releasing a buffering agent calcium into the bloodstream, activating bone resorption. With more calcium entering the bloodstream, the kidneys respond by increasing urinary excretion, resulting in a net loss of calcium. There is also a link between high-fat diets and bone loss, as fat is suggested to inhibit osteoblast formation [80,81,82,83].

### 2.8. Over-Supplementation and Physical Rehabilitation Program

Patients with liver disease commonly suffer from obesity because of continued oral intake along with limitations in physical activity that are often recommended due to fear that exertion would hasten the progress of ESLD progression or worsen complications. However, exercise is documented not to adversely affect liver function tests or worsen symptoms. In fact, the adverse effects of inactivity and bed rest may not only worsen the complications of reduced physical functioning, muscle wasting and osteopenia, but may also be linked to decreased post-LT success [83,84].

Obesity is also considered a predictor of hepatic steatosis in deceased [85] and living donors [12]. A donor’s fatty liver is strongly associated with decreased allograft function and decreased patient survival [86], and the presence of fatty liver is the main reason for the discarding of potential donor livers [85]. Pre-LT obese patients may be more likely to have primary graft dysfunction or delayed graft function after LT [87,88]. Weight loss is used to reduce the amount of liver fat among obese patients [87].

Dietitians need to resist the temptation to reach the impractical goal of producing anabolism. Attempts to replete the malnourished metabolically stressed pre-LT patient in excess of the patient’s energy expenditure lead to hyperglycemia and increased incidence of infection [48]. The goal of nutritional support for the patient with liver failure is to provide adequate protein and energy equivalent to, or slightly less than, the patient’s energy expenditure. Therefore, energy restriction to 25–30 kcal/kg/day is routinely used to encourage the mobilization of native fat stores [89].

Recently, a rehabilitation program has been introduced to encourage early postoperative mobilization and avert pulmonary dysfunction. Because LDLT is an elective procedure that differs from DDLT, a pre-LT rehabilitation program can be implemented until the day of LT [7].

### 2.9. Immunonutrition

Use an immuno-modulating diet (IMD) as a part of EN or PN is based on its down-regulatory effects on inflammatory cytokine production, its modulation of eicosanoid synthesis, and its amelioration of the necrotized liver injury and post-LT immunosuppression, rather than its effects on nutrition per se [90,91].

Glutamine dipeptide, arginine, nucleotides and omega-3 fatty acids (fish oil emulsion) intake has been suggested to minimize ischemia or reperfusion damage of the donor organ [10,63,92]. Arginine stimulates the release of growth hormone, insulin release, improves nitrogen balance, promotes wound healing, strengthens immune function and enhances nitric oxide biosynthesis [89].

An IMD enriched with hydrolyzed whey peptide (HWP), which is a protein complex derived from milk, has been proved to decrease post-LT bacteremia, infections and mortality compared with a conventional elemental diet [93]. These benefits are attributed to the antioxidant, antihypertensive, antiviral, anti-inflammatory, and antibacterial properties of HWP because it is rich in lactoferrin, β-lactoglobulin, α-lactalbumin, glycomacropeptide, and immunoglobulins [10,94]. Lactoferrin protects against the development of hepatitis caused by the sensitization of Kupffer cells by lipopolysaccharide and inhibits the production of inflammatory cytokines such as TNF-α, IL-1β, and IL-6 in monocytes [4,7,73].

The considerable amount of steroids administered to patients after LT, as well as surgical diabetes and insulin resistance, can cause intra- and postoperative hyperglycemia, which has been associated with surgical site infections [92]. IMD enriched with HWP contains isomaltulose disaccharide (glucose plus fructose with a glycosidic bond). Isomaltulose is often used instead of sugar in diets for patients with diabetes mellitus since it prevents postprandial hyperglycemia due to slow resolution. An IMD enriched with HWP has been found to significantly decrease the incidence of post-LT hyperglycemia [93,95].

### 2.10. Use of Synbiotics

Bacterial translocation usually occurring in LC is related to bacterial overgrowth, increased intestinal permeability and immune alterations, and leads to intestinal edema, decreased peristalsis, and infection. It also contributes to pathogenesis of a hyperdynamic circulatory state and multiple organ dysfunction via pro-inflammatory cytokine responses [11,96,97].

Probiotics are living bacteria found in fermented beverages, yogurt and sauerkraut that foster a hostile colonic environment against bad bacteria. Prebiotics are non-digestible dietary fiber that pass unchanged through the gastrointestinal tract and nourish probiotics. Synbiotics are a combination of both [97,98].

Sugawara et al. [99] reported that preoperative oral administration of synbiotics can enhance the immune response, attenuate the systemic postoperative inflammatory response, and decrease the occurrence of post-LT infection and the duration of antibiotic therapy. These benefits of synbiotics are attributed to the ability of *Lactobacillus* to initiate immunoglobulin production, restore macrophage function, stimulate apoptosis, and modulate lymphocyte function. In addition, *Lactobacillus* is reported to attenuate cytokine release, increase mucin production, eliminate toxins, and stimulate mucosal growth [100].

Probiotics such as Enterococcus faecalis, Clostridium butyricum, Echerichia coli strain Nissle 1917, Lactobacillus casei strain Shirota, Bacillus mesentericus, Lactobacillus, and Bifidobacterium with fructooligosaccharides can all alter gut microbiota, prevent bacterial translocation, decrease endotoxin levels and restore neutrophil phagocytic capacity [97], since neutrophil function is impaired by endotoxemia upon bacterial translocation in LC [101].

Lower ammonia levels, significant rates of minimal HE reversal and good adherence by patients, with greater improvement in all neuropsychological tests, have been all observed upon use of probiotics compared with use of conventional lactulose [102]. Furthermore, lactulose treatment was associated with occasional abdominal pain, cramping, diarrhea and flatulence. Synbiotic supplements were free of such adverse effects [102,103,104,105].

### 2.11. Nocturnal Meals

Periods of fasting should be avoided in cirrhotic patients. Frequent meals should be implemented to combat catabolic state during the overnight fasting period. Because of this, nocturnal supplementation of a small bedtime snack and nocturnal glucose supplementation increase carbohydrate along with decreased lipid and protein oxidation rates in the next morning without significant BEE changes, thus improving nitrogen balance, total body protein gain and preventing catabolic states and undernutrition [106,107]. It has been reported that nocturnal BCAAs administration as a late evening snack (LES) improves the serum albumin level and glucose tolerance in LC patients [31,106,107].

Some reports found that carbohydrate-predominant LES promote improved nutritional status [108,109]. Others reported that LES with BCAA were useful in improving protein metabolism and lipolysis in cirrhotic patients and that energy efficiency of BCAA is higher than that of glucose or carbohydrates [31,106,107].

For adult recipients preparing for LDLT, Kaido et al. [10,11] illustrated a detailed preoperative nutritional therapy regimen. It starts approximately 2 weeks before LDLT after BIA assessment. The therapy consists of the following three components: a nutrient mixture enriched with BCAAs or BCAAs nutrients as late evening nutrients; synbiotics using a supplementation product enriched with glutamine, dietary fiber and oligosaccharide three times daily, and a lacto-fermented beverage containing 5 × 10^8^/mL of *Lactobacillus casei Shirota* strain once a day via feeding tube or orally until discharge. Additionally, patients with low serum zinc level receive 1.0 g/day of polaprezinc.

Dietitians should adjust the type and amount of food for each patient to maintain a total energy intake at least 1.3 times the BEE (e.g., a protein intake of 1.2 to 1.5 g/kg including BCAAs nutrients (scaled according to degree of hepatic decompensation) adherent to ESPEN guidelines ESPEN [41,42]. Of the total non-protein energy requirements, 60–70% should be administered as high-complex and simple carbohydrates, whereas lipids should make up the other 40–50%. In malnourished patients, a daily intake of 50 kcal/kg is required for energy repletion [23]. Excess calories should be avoided, as this promotes hepatic lipogenesis, liver dysfunction and increased carbon dioxide production, leading to increased work of breathing [23,42,43].

## 3. Nutritional Support after LT

Liver disease, nutrition abnormalities are expected to correct when a new functioning liver is in place. However, unlike other complications, a reverse of malnutrition and more specifically of sarcopenia is not a rule after LT. Therefore, despite the regain of liver function after LT and the improvement in body weight after surgery, the alterations in body composition may persist. In particular, muscle depletion seems to persist for at least 12 months or more [38,39]. Moreover, other features of malnutrition, such as overweight and obesity, may occur in liver recipients during long-term follow-up.

During the stay of surgical intensive care unit (SICU), nutritional support should be emphasized on the destination of graft function recovery and overall convalescence which is faced with stress from critical illness and multiple treatments (mechanical ventilation, hemodiafiltration, use of corticosteroid and immunosuppressive agents and so on) [28,44].

### 3.1. Factors That May Influence Nutritional Modifications after LT

#### 3.1.1. Liver Gut Brain Axis

The normal hepatic innervations and more specifically, vagus innervation, are lost during transplantation. It has been suggested that the isolation of the liver from the autonomic regulatory control may influence not only nutrient absorption and metabolism, glucose and lipids homeostasis but also appetite signaling and eating behavior [104]. All of these modifications may contribute to the body composition and weight changes observed in liver transplanted patients [105,106].

#### 3.1.2. Diet

The majority of the published studies reported a significant increase in dietary intake when the patients were followed after liver transplantation. These changes are particularly evident in those patients following severe dietary restrictions or in those suffering from relevant gastrointestinal symptoms or anorexia before LT [107,108]. Calories reportedly improved from a median of 27 kcal/kg/day to 32 kcal/kg/day and proteins from a median of 0.8 g/kg/day to 1.3 g/kg/day (comparing dietary intake before LT and 12 months post-transplant [73]. Moreover, overweight or obesity in LT long-term recipients was correlated with the increase in energy intake (from 1542 ± 124 kcal/day to 2227 ± 141 kcal/day), a higher consumption of both proteins and carbohydrates and an approximately doubled intake of fat (from 62 g/day to 102 g/day) compared to pre-transplant [107].

#### 3.1.3. Immunosuppressive Therapy

Corticosteroids need attention as they increase appetite and fat deposition and decrease fat oxidation; moreover, they are responsible for increased proteolysis and impaired protein synthesis [89]. Calcineurin inhibitors, such as cyclosporine and tacrolimus, may affect energy metabolism and muscle mass [95]. Cyclosporine was found to be an independent predictor of post-transplant weight gain [89], whereas tacrolimus has been reported to increase energy expenditure [95]. Both cyclosporine and tacrolimus may contribute to the impairment of muscle growth and muscle regeneration by inhibiting calcineurin, which exerts its effects on skeletal muscle differentiation, hypertrophy, protein accretion and fiber-type determination [89,95]. Other immunosuppressive agents, such as sirolimus and everolimus, negatively influence muscle mass by inhibiting the mammalian target of rapamycin complex, which is a key regulator of protein synthesis [95].

After liver transplant, patients will have to take immunosuppressant medication to the end of their lives. Although modern drugs with less side effects are available, increased survival rates and decreased overall complications have led to many nutrition status implications associated with the use of cyclosporine, tacrolimus and corticosteroids. New onset diabetes or glucose impairment is common initially after the operation as the consequence of immunosuppressant regiment [105,106]. Diabetic dietary advice is usual required, and if necessary, the use of oral hypoglycemic or insulin regimens should be tethered according to the progression of diet. If hyperglycemia persists, it should be managed by reducing excess glucose intake, since higher insulin might hamper increased glucose oxidation in this period. Also, the diabetogenic potential of the immunosuppressant tacrolimus may be lowered by reducing its dose, without undue risk of rejection [109].

Many patients may concomitantly present with high potassium levels shortly after the operation. This usually results from the nephrotoxicity of the prescribed immunosuppressant medication. Thus, in the early post-transplant periods, it might be important to control potassium food sources as well as, it the recommendation of the use of dietary techniques which are able to reduce its content in nutrients [106]. In the long term, this is not indicated, as this condition mostly disappears. Hypomagnesemia also rises as a consequence of immunosuppression and, patients generally receive magnesium supplementation, however, some progress with diarrhea. The intake of magnesium rich food sources should be encouraged, such as dark cocoa, whole grains, nuts, legumes, fruits and green vegetables. Important to point that the consumption of this kind of food should not be restricted, even considering the immunocompromised host as a result of anti-graft rejection drugs. Patients should receive food safety advice to prevent food borne infections, which can be achieved with the correct handling of fruits and vegetables [95]. “As a result of immunosuppressive medications, the transplant recipient, in addition to other at-risk people such as those suffering from diabetes, kidney disease; infants; and the elderly—are 15–20% more susceptible to foodborne illness than the general population as shigella, yersinia, norovirus, rotaviruses, cryptosporidium, Toxoplasma gondii, Trichinella and Giardia lamblia. The commonly assumed mode of food contamination traditionally involved undercooked meats fish, poultry, eggs, fermented foods, and unwashed raw fruits, and vegetables, fresh salad dressings containing aged cheese raw, non-heat treated honey or unpasteurized dairy products [110]. Pasteurization and sufficient cooking kill Listeria; however, contamination with hepatitis A, *E. coli*, Listeria, Salmonella, may occur after cooking and before packaging by fecal contamination if handled by food handlers. Flies, may act as carriers of contamination either directly by laying eggs on the meat by transporting contaminants from one source to another. the transplant recipient may experience a more rapid onset of symptoms compared with the general population, in the form of severe dehydration and organ failure/hemolytic uremic syndrome (*E. coli*), sepsis, or death (Listeria and Clostridia botulinum). Prevention of foodborne illness involve thorough cleaning, hand washing, avoiding raw meat or canned food, maintaining appropriate storage temperature [7,110]. Grapefruit, turmeric, pomelo, ginger, pomegranates, Seville oranges, black pepper, cranberry juice, black tea, beer, cruciferous vegetables, kava, licorice root, wine, and olive oil, contain compounds that modulate P450 activity juice should be avoided as elevate blood immunosuppressant’s levels. Other interactions to be avoided include K-rich foods (e.g., broccoli, spinach) and Coumadin. Foods containing the substance tyramine, including chocolate, beer, wine, avocados, some aged cheeses, and some processed meats, and monoamine oxidase (MAO) inhibitors, a type of antidepressant result in a dangerous rise in blood pressure. Natural licorice, which contains the compound glycyrrhizin, can reduce the effectiveness of blood pressure medications and diuretics such as Aldactone (spironolactone). It can also increase the risk of Lanoxin (digoxin) toxicity. Resveratrol, an antioxidant compound found in red wine and peanuts, inhibits platelet aggregation, and high intakes could increase the risk of bleeding when consumed with anticoagulant drugs such as Coumadin [111].

#### 3.1.4. Energy Metabolism

Hypermetabolism after transplantation was significantly associated with hypermetabolism before LT and a higher cumulative dose of prednisone. Energy expenditure normalized only when insulin sensitivity was restored. However, those patients with a reduced energy expenditure showed the higher increase in fat mass [107,108,109].

After the LT operation, energy and protein requirements are still increased for weeks. Metabolism in liver recipients only improves at 4 weeks after LT, especially considering the non-protein respiratory quotient, serum non esterified fatty acids and nitrogen balance [109]. In the immediate phase after the operation, protein catabolism is markedly increased and patients should receive about 1.5–2.0 g/kg of protein. Non-protein energy requirements, in this period, vary according to the metabolic and inflammatory status, with unstable patients demanding lower intakes while the others more. When indirect calorimetry is not available, estimates between 25 and 30 kcal/kg/day maybe used [108,109].

Total body water decreases and body fat increases, whereas BCM remains unchanged after LT [107]. Deficiencies in vitamin A and zinc immediately normalize after LT [108,109]. Increased REE may persist for a long period after LT [108,112]; however, overweight status and hypercholesterolemia have been observed after LT [110,111], accompanied by an increase in the saturated fatty acid content of fat tissue [109,113].

Nutritional status after LT depends on the allograft function; if the allograft fails or is rejected, many of the nutritional derangements present before LT will persist. Even in a well-functioning graft, some nutritional disturbances do not completely normalize in the long term after LT. Increased protein breakdown is often present during the first 2 weeks post-LT; thus, optimizing the nutrient intake over this period is needed for wound healing and hepatocyte recovery [109,114].

### 3.2. Nutritional Support during the Immediate Post-LT Phase and Short-Term after LT

The goal of nutrition therapy in the acute post-LT phase is to ensure adequate protein and energy provisions to avoid protein breakdown [115]. Hypermetabolism has been found predictive of transplant-free survival independently of MELD and Child-Pugh scores and tends to persist for at least a year post-LT [116].

Resuming EN within 12 h of LT has been shown to reduce postoperative viral infections and to produce better nitrogen retention. Patients should be advanced from nutritional support to an oral diet using smaller and more frequent feedings as soon as tolerated after LT. EN should not be discontinued until patients are able to maintain an adequate oral intake consistent with their nutritional requirements [10,11,94].

Intra-operative placement of the tip of the feeding tube in the proximal jejunum allows early EN after LT. For adult LDLT recipients, Kaido et al. [10,11,94] described in detail an early postoperative EN regimen using a 9F Witzel enteral tube jejunostomy placed in the proximal jejunum at surgery, through which an EN is started within 24 h after surgery. The starting total daily energy intake until postoperative day (POD) 3 is 10–15 kcal/kg and gradually increased to 25–35 kcal/kg using an IMD enriched with HWP. The initial infusion rate is 20 mL/h. If well tolerated, it is increased to 40 mL/h by POD 5. In case of severe edema of the small intestine or severe diarrhea, the speed of IMD is decreased to 20 mL/h (=20 kcal/h) or an oral rehydration solution is used. After confirmation of improvement in edema or diarrhea, the regimen can be resumed. Oral nutrition is started after swallowing ability is confirmed, usually around POD 5. Dietitians calculate the daily amounts of protein and carbohydrates required for each recipient and adjust the speed of the EN according to the oral intake. EN is stopped when the patient can tolerate adequate oral intake containing solid food. All patients resume preoperative synbiotic supplementation three times daily and a lactic fermented beverage once a day via the feeding tube or orally until discharge. This technique allows long-term feeding without discomfort or risk of pneumonia carried by trans-nasal feeding and avoids the need for concomitant TPN with risk of infection.

Metabolic alkalosis and depletion of serum potassium, phosphorus, and magnesium levels in the acute post-LT period due to routine chronic diuretic use in cirrhotic patients, amount of fluid from abdominal drains, gastrointestinal losses or fluid overload should be monitored. Also, refeeding syndrome should be taken as a risk factor for these disorders. In cases of metabolic alkalosis, 90% are chloride-sensitive and easily correctable. Chloride can be delivered using TPN as a vehicle [9,10,11,117].

Glucose utilization by the transplanted liver is reduced in the first hours of engraftment, due to impaired mitochondrial respiration and inactivity of the tricarboxylic acid cycle [118,119]. During this time, energy is generated mostly from fatty acid oxidation and after approximately 6 h, a shift from fat to glucose utilization occurs in normally-functioning liver grafts, while a failing liver continues to utilize mainly fat [119,120]. Glucose administration immediately after LT has been recommended in small quantities and without insulin in order not to suppress peripheral fat mobilization, judged clinically by blood glucose, lactate, triglyceride levels and arterial ketone bodies [119,120,121,122].

Diabetic patients with liver failure receiving EN should be covered with long-acting isophane insulin suspension on a sliding scale for episodes of hyperglycemia [123].

Patients with fulminant hepatic failure are generally well nourished and do not have a pre-hospital history of weight loss. Patients without PEM will tolerate 5–7 days of NPO before needing nutritional support, however, an adequate nutritional supplementation is required as early as comatose patients are put on liver dialysis and on through to support regenerating hepatocytes. Patients with malnutrition should start nutritional support sooner. Withholding nutritional support and inducing a cumulative energy deficit of over 10,000 kcal has been associated with decreased survival [89].

### 3.3. Long-Term Nutritional Support after LT

In the long-term after liver transplantation, weight gain is mostly observed. It is important to recover the nutritional status, since the patients lose an average of 9.1 kg during the course of liver disease [117]. Greatest relative weight gain occurs in the first six months after the operation [47] and, recovery of all weight loss happens in the first post-transplant year [124]. However, unfortunately, patients do not stop gaining weight in the subsequent years [125], resulting in the alarming prevalence of overweight and obesity [47]. During the first 12 months, the fat mass progressively increases in those patients who had previously depleted overall body mass, but muscle mass recovery is subtle and non-significant by the end of the first year [126]. Therefore, despite the weight gain, the high prevalence of sarcopenia does not change after transplantation [7,47].

Metabolic syndrome, hyperlipidemia and obesity are common in patients after the first 6 months post-LT, especially with immobility, and is associated with an increased risk of major vascular events, diabetes mellitus, hypertension, cancer and fibrosis progression. These conditions contribute to long-term morbidity and mortality [7,47,117,124].

Weight gain is mostly between 2 and 16 months after LT, attributed to stimulated appetite by corticosteroids. Immediately after LT, patients are often instructed to ingest a high-protein, high-energy diet to counteract weight loss associated with pre-LT cachexia and increased energy requirements for surgical recovery but can induce unwanted weight gain. Depressive moods have been implicated in over- and under-eating and should be considered a factor in LT recipients; therefore, patients should be instructed on a diet that promotes a healthy body composition which is low in fat, with adequate amounts of lean protein foods to promote muscle gain. Calories should be sufficient to spare protein from being used as energy, yet not in excess of energy requirements. Regular follow-up with a dietitian will ensure patient compliance. Dietitians should re-assess nutritional status to optimize the patient’s diet during the transition from the acute to chronic post-LT phase [41,49,125,126,127].

Tacrolimus is thought to be associated with a less adverse cardiovascular risk profile than cyclosporin, with significantly reduced prevalence of hypertension, hypercholesterolemia and obesity, together with significantly lower triglyceride levels. Corticosteroids also contribute to post-LT disturbances of these parameters. In patients with stable graft function, withdrawal of prednisolone over time reduces prevalence of such disorders [117,128]. Long-term administration of glucocorticoids results in lipid accumulation, weight gain, osteoporosis and muscle-wasting by impairing REE and substrate oxidation rates. Insulin resistance, postoperative cytokine response, and postmenopausal status in women are other suggested mechanisms that inhibit gain of muscle mass after LT [129].

Standard recommendations after LT include a “no added salt” diet (3 g sodium/day) to prevent water retention associated with steroid therapy. However, health professionals often encourage the addition of flavoring agents, including sodium, to foods to improve taste in order to promote appetite. Therefore, sodium intake may be higher than suspected [89].

**Figure 4:**
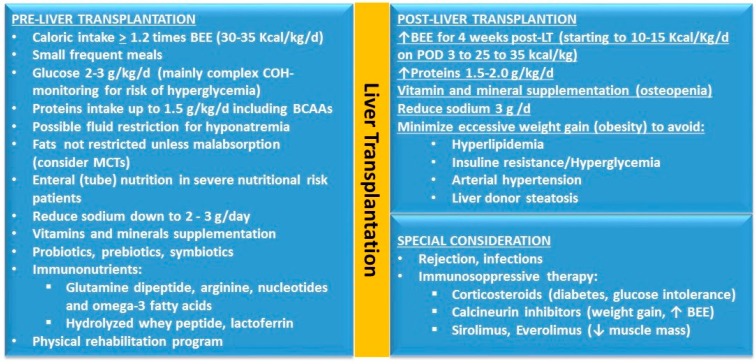
Nutritional interventions before and after liver transplantation. BEE: basal energy expenditure; BCAA: branched chain amino acids; COH: carbohydrates; MCTs: mean chain triglycerides; LT: liver transplantation; POD: post operative day.

Several risk factors for bone loss after LT include steroid use, malnutrition, muscle-wasting, immobilization, pre-LT osteopenia or osteoporosis, previous fractures, and immunosuppressive agents. Bone loss occurs mostly within the first 3–6 months after LT and increases the risk of fractures within the first year. However, osteopenia related to cholestasis tends to become stable at 1 year after LT following improved allograft function. Bisphosphonates may prevent bone loss after LT [78,130,131,132,133,134,135] (Figure 4).

## 4. Special Considerations in Pediatric LT

In children, malnutrition and growth retardation are usually present in all cases before LT, specifically linked to the most common indication; biliary atresia. Partial substitution of usual fats with medium chain triglycerides, and carefully monitored supplementation of fat soluble vitamin are needed. Anthropometry derangement starts to recover as soon as 6 months after LT. Height recovery occurs late [128]. Marked catch-up growth is observed in those children with the most severe growth retardation before LT. However, transplanted children do not have complete catch up growth and achieve a final height below their genetic potential and even some children experience failure to thrive after LT [110].

Although individually rare, when considered together, liver-based metabolic diseases represent approximately 10% of pediatric liver transplants [136]. Strict galactose- or fructose-free preoperative diets are needed in galactosemia and hereditary fructose intolerance, respectively. Frequent feedings are needed in glycogen storage diseases, which often includes continuous nighttime nasogastric feedings in infants. Uncooked cornstarch ingested every few hours in older patients has been shown to release glucose slowly and steadily and allows avoidance of hypoglycemia [137]. In tyrosinemia, tyrosine free-diet and Nitisinone (NTBC), which blocks the second step in tyrosine degradation are fundamental [138].

Medical treatment during the acute presentation of urea cycle disorders is based initially on reducing blood ammonia levels by (a) discontinuing protein intake and supplying sufficient glucose intravenously to limit catabolism (b) providing biochemical alternatives for nitrogen excretion as intravenous or oral sodium benzoate and phenylacetate [138,139]. Long-term dietary protein restriction is paramount. Enzyme defects block synthesis of arginine and overall protein-restricted diet will lead to arginine deficiency. Arginine supplementation is therefore essential in argininosuccinic aciduria or argininemia to increase nitrogen excretion. However, in carbamyl phosphate synthetase or ornithine transcarbamylase deficiency, citrulline supplementation is preferred [137,138,139].

In maple syrup disease, dietary restriction of BCAA and aggressive management of episodic metabolic decompensation are required. The B12 supplements are added for methylmalonic aciduria. Tin-protoporphyrin or zinc-mesoporphyrin may decrease the hours of phototherapy required per day or the need for exchange transfusions in crigler najjar cases. Hydrophilic bile acids are used for erythropoietic protoporphyria. Copper-free diet and zinc salts supplements when used in a timely manner can augment chelation therapy to prevent progression of Wilson disease. Likewise, iron-free diet in haemochromatosis. Mannose or Fucose supplementations are essential in some congenital disorders of glycosylation. Dextrose 10% infusion suppresses heme synthesis in porphyria [137,138,139]. A further comprehensive and detailed discussion of age-specific nutritional treatment in pediatric LT is warranted.

## 5. Conclusions

Nutritional therapy should be considered an essential adjunct to clinical therapies, especially when the patient is a candidate for LT. Accurate assessment of nutritional status and adequate intervention are prerequisites for perioperative nutritional treatment in patients who undergo LT. However, the metabolic abnormalities induced by liver failure cause the traditional assessment of nutritional status to be difficult. Preoperative malnutrition and sarcopenia estimated by recently-developed body BIA have a significant negative impact on post LT outcome. It is essential to provide adequate nutritional support during all phases of liver transplantation, including preoperative administration of BCAA-enriched nutrient mixture and postoperative use of an IMD enriched with HWP. Perioperative nutritional therapy is now indispensable to improve outcomes after LT. Further studies are warranted to refine patient-tailored nutritional regimens and optimize nutritional recovery and rehabilitation long-term after LT.
