# Correction: Hammad, A.; Kaido, T.; Aliyev V.; Mandato C.; Uemoto S. Nutritional Therapy in Liver Transplantation. Nutrients 2017; *9*. E1126

**DOI:** 10.3390/nu10122006

**Published:** 2018-12-18

**Authors:** Ahmed Hammad, Toshimi Kaido, Vusal Aliyev, Claudia Mandato, Shinji Uemoto

**Affiliations:** 1 Division of Hepato-Biliary-Pancreatic and Transplant Surgery, Department of Surgery, Graduate School of Medicine, Kyoto University, Kyoto 606-8501, Japan; ahmedhammad2005@yahoo.com (A.H.); dr.vusal@outlook.com (V.A.); uemoto@kuhp.kyoto-u.ac.jp (S.U.); 2 Department of General Surgery, Mansoura University, Mansoura 35516, Egypt; 3 L’AORN Children’s Hospital Santobono and Pausilipon, Napoli 80122, Italy; cla.mandato@gmail.com

The authors wish to make the following correction to the published version of the paper [1], which had incorrect bibliographic information in the text, citing a few paragraphs of reference [2] (Anastácio, L.R.; Correia, M.I.T.D. Nutrition therapy: Integral part of liver transplant care. *World J. Gastroenterol.* **2016**, *22*, 1513–1522). The information has been corrected by the authorship team.

The last paragraph on p. 13 and the first one 14: “After liver transplant, patients will have to take immunosuppressant medication to the end of their lives. Although modern drugs with less side effects are available, increased survival rates and decreased overall complications have led to many nutrition status implications associated with the use of cyclosporine, tacrolimus and corticosteroids. New onset diabetes or glucose impairment is common initially after the operation as the consequence of immunosuppressant regiment [105,106]. Diabetic dietary advice is usual required, and if necessary, the use of oral hypoglycemic or insulin regimens should be tethered according to the progression of diet. If hyperglycemia persists, it should be managed by reducing excess glucose intake, since higher insulin might hamper increased glucose oxidation in this period. Also, the diabetogenic potential of the immunosuppressant tacrolimus may be lowered by reducing its dose, without undue risk of rejection [109].

Many patients may concomitantly present with high potassium levels shortly after the operation. This usually results from the nephrotoxicity of the prescribed immunosuppressant medication. Thus, in the early post-transplant periods, it might be important to control potassium food sources as well via the recommendation of the use of dietary techniques that are able to reduce its content in nutrients [106]. In the long term, this is not indicated, as this condition mostly disappears. Hypomagnesemia also rises as a consequence of immunosuppression and, patients generally receive magnesium supplementation, however, some progress with diarrhea. The intake of magnesium rich food sources should be encouraged, such as dark cocoa, whole grains, nuts, legumes, fruits and green vegetables. Important to point that the consumption of this kind of food should not be restricted, even considering the immunocompromised host as a result of anti-graft rejection drugs. Patients should receive food safety advice to prevent foodborne infections, which can be achieved with the correct handling of fruits and vegetables [95]”.

These two paragraphs are corrected to read: “After liver transplant, patients will need immunosuppressive medications for lifetime. Although new drugs with less side effects are available, increased survival rates and decreased overall complications have caused multiple nutritional implications linked to the use of cyclosporine, tacrolimus or corticosteroids [110]. New onset diabetes mellitus or glucose intolerance is not uncommon short after the operation as the consequence of immunosuppressant treatment [105,106,110]. Diabetic dietary recommendation is usually needed, and if required, the use of oral hypoglycemic or insulin treatments should be tailored according to the advancement of diet. If hyperglycemia remains, it should be addressed by decreasing glucose intake, since higher insulin might prevent increased glucose oxidation during this period. Also, the possible diabetogenic effect of tacrolimus may be decreased by decreasing its dose, without added risk of rejection [109,110].

Many patients may concurrently have high potassium levels shortly after transplant [110]. This is usually due to the nephrotoxicity of the used immunosuppressive drug. So, it might be crucial to adjust potassium food sources during the early post-operative period, and use of dietary modifications which would decrease its content in nutritional intake [106,110]. This is not required long-term after transplant, as this transient imbalance often disappears. Hypomagnesemia also occurs due to immunosuppressants and, patients usually have magnesium supplements, however, some will have diarrhea. Magnesium-rich food intake should be carried on, such as dark cocoa, whole grains, nuts, legumes, fruits and green vegetables [110]. It is crucial to mention that consumption of such kind of food should not be restricted, even considering the immunocompromised host as a result of anti-graft rejection drugs. Patients should receive dietary safety recommendations to avoid food borne infections, with the correct handling of fruits and vegetables [95,110].

The first paragraph in Section 3.3 on p. 16: “In the long-term after liver transplantation, weight gain is mostly observed. It is important to recover the nutritional status, since the patients lose an average of 9.1 kg during the course of liver disease [117]. Greatest relative weight gain occurs in the first six months after the operation [47] and, recovery of all weight loss happens in the first post-transplant year [124]. However, unfortunately, patients do not stop gaining weight in the subsequent years [125], resulting in the alarming prevalence of overweight and obesity [47]. During the first 12 months, the fat mass progressively increases in those patients who had previously depleted overall body mass, but muscle mass recovery is subtle and nonsignificant by the end of the first year [126]. Therefore, despite the weight gain, the high prevalence of sarcopenia does not change after transplantation [7,47].”

This paragraph has been changed to “Weight gain is generally seen long-term after liver transplantation [110]. It is a priority to recover the nutritional status, since the patients lose an average of 9.1 kg during the course of liver disease [110,119]. Largest relative weight gain occurs in the first 6 post-operative months [47,110] and, recovery of all weight loss happens within the first post-transplant year [110,125,126]. However, patients do not stop gaining weight in the subsequent years [110,127], resulting in overweight and obesity high prevalence [47]. During the first year, the fat mass progressively increases in those patients who had previously depleted overall body mass, but muscle mass recovery occurs at a relatively lower rate by the end of the first 12 months [128]. Hence, despite the ongoing weight gain, the high prevalence of sarcopenia does not decrease after LT [7,47,110].”

The authors apologize for any inconvenience caused to the readers of *Nutrients* by these changes. The changes do not affect the scientific results. The original manuscript will be updated and will remain online on the article webpage, with a reference to this correction.
